# Exploring Consumer Perception of Food Insecurity Using Big Data

**DOI:** 10.3390/foods14172965

**Published:** 2025-08-25

**Authors:** Hyosun Jung, Hye Hyun Yoon, Meehee Cho

**Affiliations:** 1 Center for Converging Humanities, KyungHee University, Seoul 02447, Republic of Korea; chefcook@khu.ac.kr; 2 College of Hotel and Tourism Management, KyungHee University, Seoul 02447, Republic of Korea; hhyun@khu.ac.kr

**Keywords:** food insecurity, social media, big data, South Korea

## Abstract

This study investigated consumer perception of food insecurity by refining data collected from social media platforms. Text mining and TF-IDF were used to extract core keywords closely related to food insecurity and analyze their meanings. In addition, time series analysis and sentiment analysis were used to examine temporal and emotional changes. The analysis results showed that keywords, such as health, stress, mental, and depression, appeared frequently, indicating that food insecurity is closely related to psychological and mental problems. In addition, consumers showed high emotional sensitivity to essential nutrients, such as vitamin D, magnesium, calcium, and omega. Furthermore, stress indices and mental and physical response indices increased simultaneously during this period, indicating that food insecurity is a factor that causes emotional and physical responses. The results of the sentiment analysis showed that negative emotions (anxiety, fear, and sadness) were higher than positive emotions, suggesting that discussions related to food insecurity have a negative emotional impact.

## 1. Introduction

Food insecurity is a fundamental problem that directly impacts human survival [1] and is considered a key indicator of structural social inequality and poverty [2]. In particular, with global crises, such as the COVID-19 pandemic, the war in Ukraine, and climate change, instability in the food supply chain is increasing, and as a result, the problems of reduced food accessibility, rising food prices, and nutritional imbalances in vulnerable groups are becoming more severe internationally [3,4,5]. Korea is no exception, and chronic or temporary food insecurity has recently emerged as a social problem, especially among single-person households, low-income families, children, and the elderly. Despite being a relatively high-income country, 4.3% of South Koreans experienced food insecurity in the first year of the COVID-19 pandemic [6,7]. Previously, food insecurity has been measured through national statistics gleaned from surveys on household and dietary trends and interviews [8,9], but these methods are hindered by the limited diversity of survey subjects, response subjectivity, and above all, the difficulties inherent in documenting trends in real-time. In addition, since the distribution, diversity, personal demographics, and dynamic characteristics of the data may not be reflected by these traditional methods, it is necessary to expand sample sizes, collection scope, and dynamic tracking to reflect consumers’ feelings about food insecurity more truthfully and objectively [10,11]. Accordingly, recently, analyzing voluntarily submitted data on the Internet, such as search terms, social media posts, and mentions in online communities, has been attracting attention as a big data-based approach to detecting social problems [12,13]. In South Korea, large online platforms, such as Naver and Daum, are used for daily information searches [14,15], and users actively use such sites to obtain information on economic difficulties, food price issues, free meals, and food support systems. Accordingly, search terms related to food insecurity carry more meaning than simple information searches and can be considered digital signals that reflect social crisis situations or the needs of vulnerable groups in real time [16]. Moreover, since food insecurity is not simply a matter of supply shortage but is also linked to psychological anxiety, it would be meaningful to be able to identify anxiety levels through data analysis on social media. By analyzing increases and decreases in the volume of search terms, related keywords, and emotional (positive and negative) trends, there is the possibility of achieving the early detection of deepening social food insecurity, regional bias, and changes in policy demands. In a study using unstructured data, Koren et al. [17] visualized the spread of regional food insecurity issues using data from Twitter (now X), and Tamasiga et al. [18] also reported that big data analysis could be used to find solutions to food insecurity and security. However, in South Korea, there has been little analysis of food insecurity based on online search terms, and systematic attempts to reflect up to date data are particularly rare.

In light of this, this study tracked the flow of search terms related to food insecurity on Naver and Daum during 2024 and empirically investigated how awareness of this topic formed and spread in domestic society through these platforms. In addition, by analyzing the correlation between major keywords, related word networks, and search patterns by period and event, it aims to provide empirically based data that can be referenced in national policies, local government welfare, and private organization support systems. This research has academic and social significance in that it explores the possibility that unstructured data can contribute to the design of food welfare policies in a digital society. It is structured as follows. The first section elucidates recent research and big data analysis trends related to current food insecurity, and the next section presents the research design, including the data used and the methodology adopted. The results section highlights the main research findings, and the conclusion outlines implications, limitations, and areas of potential future research.

## 2. Related Studies

In efforts to tackle the problems facing modern society, achieve secure competitiveness, and promote sustainable growth, big data research has moved from the periphery to become a core strategic field [19]. This is because decision making, which once depended on intuition or experience, is now based on quantitative and scientific evidence gleaned through big data analysis [20]. Big data refer to data that are so large in volume, high in speed, and diverse that they are difficult to collect, store, manage, and analyze using existing data analysis methods [21]. Big data are not only large but also complex, generated at high speeds, and exist in various forms [22].

Since social media networks are characterized by the 5Vs of big data (velocity, volume, value, variety, and veracity), big data analysis techniques and frameworks are widely used in their analysis [23]. Traditionally, data on users’ interests and behaviors have been collected through questionnaires, and this method is still an important tool in social science, but the emergence and popularity of social networks have made it possible to collect data on user behaviors in an unprecedented way [24]. Fan et al. [25] pointed out that the rich information provided by big data has triggered the development of new statistical and computational paradigms, enabling more sophisticated scientific discoveries. In this way, big data is also being used as a tool for solving social problems, especially in the public sector, by eliminating uncertainty and improving the quality of decision-making [26].

The following are big data studies on food insecurity, which can be considered a representative keyword in the public sector. Balashankar et al. [27] extracted text features based on news articles from 1980 to 2020 and found that the risk prediction for food insecurity can be improved compared to existing ones, and Ahn et al. [28] developed keywords to predict hunger by analyzing 53,000 news articles from 9 African countries. In addition, Eskandari et al. [29] investigated conversations related to food poverty on the social media platform Twitter during the early and later stages of the COVID-19 pandemic and found that individuals’ tweets overwhelmingly contained opinions about the increase in hunger, food poverty, and food insecurity due to the pandemic. In addition, they highlighted the rapid increase in food poverty due to hoarding, food shortages, decreased food purchasing power, and the collapse of food supplies and food systems. From a similar perspective, Martin et al. [30] analyzed Twitter in 2020 and 2021 and found that mentions of food stores, food banks, and emergency preparedness increased after the declaration of the COVID-19 pandemic. Bartelme et al. [31] focused on food poverty discourse on Twitter in Germany and presented a situation in which personal and public food insecurity experiences during the COVID-19 period were shared in real time on social media. Goetz et al. [32] also predicted emotion-based indicators, analyzing 1.2 million tweets in the United States in 2020 and proving that emotion indicators, such as fear and anger, were significantly correlated with weekly food shortage rates. Lukyamuzi et al. [33] used data mining to track tweets pertaining to food insecurity in Uganda and were able to extract relevant information and allow mitigation against it.

## 3. Research Methodology

### 3.1. Data and Summary Statistics

This paper investigated keywords related to food insecurity on social media and sought to identify changes in trends following COVID-19. The collection of data was limited to blogs and “cafes” (a kind of social media forum/group) on the South Korean-based social media platforms Naver and Daum, as it was not possible from private Facebook or Instagram accounts. Naver and Daum blogs and cafes are popular platforms in South Korea, with their members sharing news from various media outlets and actively engaging in community and forum activities. Therefore, they possess the most up-to-date content. Instagram and Facebook were excluded because many of their features do not allow private access. Therefore, it was determined that collecting keywords related to food insecurity through cafes and blogs on the two platforms would be an effective method. The data used in this study were collected from 1 January to 31 December 2024 using the keyword “food insecurity”.

### 3.2. Methodology

This involved examining consumer perceptions of food insecurity after COVID-19 by refining data collected from online social media platforms. The keywords for data search research were selected by domain experts, who clarified the purpose of data analysis and the relevance of the keywords. Data were collected through IMC, a big data company, and the TEXTOM analysis tool (Daegu, South Korea) was used for extraction and analysis. The keywords were categorized by frequency, and Ucinet 6.0 was used to analyze meaningful relationships between them. In addition, core keywords were derived, and their meanings were analyzed through text mining and term frequency-inverse document frequency (TF-IDF). In order to analyze the interrelationships between major keywords, a network was developed based on the co-occurrence of two keywords in the same document, and the connection strength and centrality values between them were confirmed. Clustering was performed to create word groups, and the network between groups was visualized. Finally, sentiment analysis was performed to identify the correlation between positive and negative emotions associated with food delivery-related keywords.

## 4. Results

### 4.1. Content Analysis

As a result of searching data with the keyword “food insecurity”, a total of 38,255 keywords were retrieved in 2024 (see Table 1). Since data collection based on TexTOM generally reports that 1000 words per channel are appropriate, the number of keywords collected in this study was sufficient. In addition, the results of a morphological analysis showed that there were a total of 10,745 words with a frequency of 10 or more. The narrative coding for food insecurity was clustered based on four criteria, namely food/nutrition, cause/need, sentimental/response, and connection/subject (Table 2).

### 4.2. Text Mining Analysis

The frequency analysis of keywords in documents extracted using the keyword “food insecurity” (Table 3) show that “food” was the most frequent keyword, followed by “insecurity”, “health”, “intake”, “stress”, “help”, depression”, “symptom”, “good”, and “mental”, demonstrating their importance in posts related to food insecurity. In addition, the TF-IDF values of keywords, such as “eat”, “effect”, “magnesium”, “brain”, “mind”, and “vitamin A” were significantly higher than other keywords, which means that they are meaningful despite not appearing frequently. TF-IDF plays an important role in short-term trend analysis by considering both the frequency of words and the irregularity of word appearance between documents, so it is expected that these keywords will be important in the food insecurity trend in 2024. It can be assumed that this was a major factor.

### 4.3. Sentimental Network Analysis

Based on the text mining analysis results, keywords related to food insecurity were analyzed using an emotional network index. The location and role of each node were analyzed using the semantic network index, and the attributes of words with high relevance were identified. The first attribute is the degree centrality, showing the closeness of the relationship between a variable and other variables. Therefore, this index can be interpreted as a factor that directly affects consumers’ emotions. The second is betweenness centrality, which shows the mediating role that a variable plays when other variables appear. Therefore, this index can be seen as a factor that greatly depends on consumers’ perceptions of emotions. The third is closeness centrality, which indicates a variable’s synergistic emotional interactions with other variables. The fourth is page rank, which shows the page rank value of a specific variable, indicating its relative popularity compared with other variables in terms of consumer emotions. Table 4 presents the results of the semantic network analysis connecting food insecurity and emotions. Consumer sentiment on food insecurity in 2024 was examined based on the degree of centrality, betweenness centrality, closeness centrality, and page rank value.

**Figure 1:**
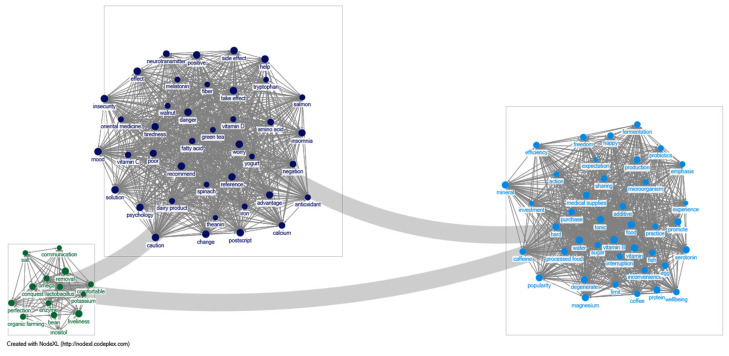
Sentimental network visualization of food insecurity.

In relation to emotions about food insecurity, this study confirmed that discourses were formed through keywords, such as anxiety, fatty acids, vitamin D, calcium, food, processed foods, magnesium, omega, and beans. In particular, it was noteworthy that keywords with high values in the emotional analysis results were essential vitamins and minerals, such as vitamin D, calcium, and magnesium. This result shows that South Korean consumers in 2024 were involved in a discourse on their anxieties about consuming essential vitamins and minerals that stemmed from their emotions related to food insecurity. It is believed that people tend to seek to protect themselves by eating healthy nutrients as their anxiety about food increases. This appears to reflect the trend of social obsession with immunity that has continued since the pandemic. The results of the visualization were divided into three categories, namely insecurity, food, and omega (Figure 1).

### 4.4. Time Series Analysis

**Figure 2:**
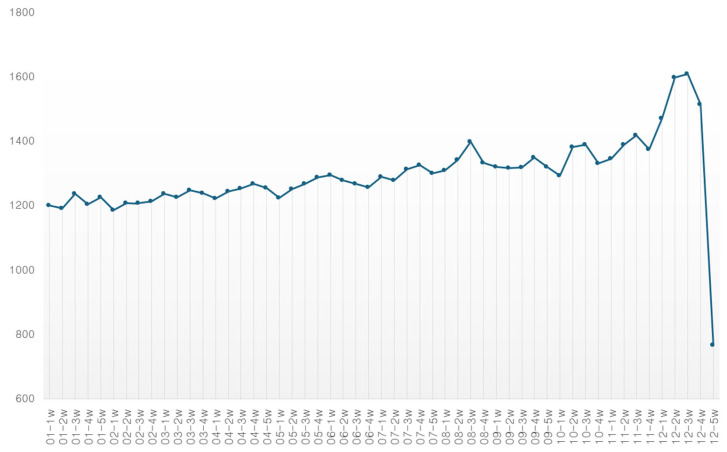
Time series analysis of food insecurity.

The results of the time series analysis of the weekly document volume on food insecurity are shown in Figure 2. In 2024, an average of 1300 food insecurity keywords were created per week, showing a sharp increase at the end of the year, followed by a decrease. It can be inferred that concerns were high enough to maintain at least 1000 mentions weekly. As a result of analyzing the volume of each word related to food insecurity, the frequency of food, insecurity, and health showed a high number of mentions throughout the previous month; it peaked in July and August but showed a sharp decrease in late December.

**Figure 3:**
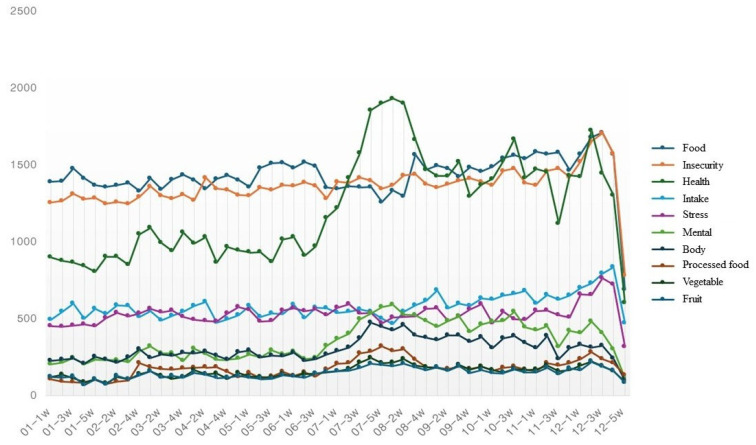
Time series analysis of key word frequencies.

The results of the time series analysis are shown in Table 5 and Figure 3. A time-series analysis was conducted to identify key words related to food insecurity by frequency. They show that the stress index also increased overall during the period from May to December when food insecurity increased. This can be interpreted as showing a tendency for stress levels to increase as food insecurity increases. As a result, it can be said the former reflects the emotional and psychological consequences of the latter. In addition, the mental and body indices also increased simultaneously during this period, suggesting that prolonged food insecurity led to mental instability and physical reactions. Processed foods also showed a continuous increase in the second half of the year, implying potential alternative food consumption patterns increasing among low-income classes.

### 4.5. Sentiment Analysis

Sentiment analysis is a field of text mining technology that analyzes subjective data, such as people’s attitudes and tendencies expressed in text. It refers to natural language processing technology that analyzes subjective data, such as people’s attitudes, opinions, and tendencies expressed in text. It has the advantage of being able to automatically and quickly process polarity analysis of linguistic expressions through sentiment analysis. Words were classified using TexTOM’s emotional categories, and then frequency and emotional intensity were calculated. As a result of analyzing keywords related to food insecurity in 2024 (See Table 6), it was found that the sub-emotions (sadness, disgust, fear, pain, anger, and fright) in the negative range (57.56%) were relatively higher than the sub-emotions (feeling good, joy, and interest) in the positive range (45.4%).

## 5. Discussion

This study derived words closely related to the keyword “food insecurity” based on social media big data and investigated related factors. The following conclusions were drawn from the results of text mining, time series analysis, and emotional network analysis of documents related to food insecurity during 2024. It was confirmed that this is a multidimensional social problem that goes beyond simple food accessibility issues and encompasses mental health, nutrient consumption, and emotional responses. In particular, keywords, such as health, stress, mental, and depression, appeared with high frequency, which means that food insecurity is directly related to psychological and mental problems beyond simple nutritional deficiencies. In addition, the results of TF-IDF and the emotional network analysis showed that consumers’ emotional sensitivity to essential nutrients, such as vitamin D, magnesium, and calcium omega was high. This can be interpreted as showing that social interest and anxiety about immunity influenced the discourse on food insecurity. In addition, stress and mental and physical response indices also increased simultaneously during this period, which shows that food insecurity is a factor that causes emotional and physical responses. In addition, this period saw an increase in processed food consumption, which can be seen as indicating the possible seeking out of alternative eating strategies among groups with unstable incomes. The results of the sentiment analysis also showed that negative emotions (anxiety, fear, and sadness) were higher than positive emotions, indicating that discussions related to food insecurity have a negative emotional impact.

For this research, we analyzed consumer discourse related to food insecurity from various angles, thereby providing a theoretical basis for interpreting this issue beyond the simple issue of food accessibility and in a psychological, emotional, and sociocultural context. First, the findings empirically supported the fact that food insecurity is not simply a physical deficiency but that it also acts as a trigger for mental stress and anxiety. It was empirically confirmed that food insecurity is not a mere environmental constraint but that it causes continuous emotional stress, which functions as a major variable affecting psychological and physical health. This suggests that food insecurity can be treated as a precursor or moderator of mental health in future social psychology research. Second, it was confirmed that emotionally-centered keywords for specific micronutrients, such as vitamin D, magnesium, and calcium, are deeply related to food insecurity. It theoretically showed that nutrient intake is a phenomenon motivated by social and emotional demands, not simply a physiological need. Ultimately, it suggested that consumers seek to secure emotional stability and a sense of control over health recovery through specific nutrients, and that changes in health awareness centered on nutrients are an axis of food insecurity, especially in South Korea. In addition, the strengthened social discourse centered on immunity after the COVID-19 pandemic is also reflected in the emotional response to food insecurity, suggesting that food consumption is no longer a matter of survival but an expression of identity and anxiety. Third, the centrality index derived through emotional network analysis showed that certain keywords, such as anxiety, nutrients, and processed foods, were not simply mentioned with high frequency, but were instead nodes that played a key role in the connection, mediation, and diffusion of emotional information. This can be said to have contributed to improving the theoretical validity of emotion-based semantic network analysis by overcoming the limitations of existing consumer discourse analysis that relied solely on word frequency. Fourth, consumers responded more strongly to negative emotions related to food insecurity, confirming that in situations of food insecurity, consumers tend to show emotional and preventive consumption behavior based on the fear of losing health rather than rational choices to gain health. This enabled theoretical expansion by strengthening the emotion-based risk perception model in future consumer behavior studies.

As a practical implication, this study suggests that policies connected to food production should go beyond simple supply expansion and include support for emotional health and education on nutrition to ensure their effectiveness. In particular, in the midst of a prolonged economic recession and high prices, it will be necessary to design policies that consider both psychological recovery and the stability of their dietary habits for the socially disadvantaged. In addition, since consumers are sensitive to essential nutrients, a strategy that links them to keywords, such as psychological stability, immunity, and mental health, is necessary when promoting functional foods. In addition, the close relationship with major nutrients means that consumers are not simply anxious about food insecurity but are also acting in a way that seeks to control their anxiety. This means that it is evolving from a problem of avoidance to a problem of substitution, supplementation, and reassurance, which provides important insights into policies related to food insecurity. In addition, from a communication perspective, public institutions and companies should include emotional messages that relieve anxiety about food beyond simply providing information that it is rich in nutrients. From a methodological perspective, it will be necessary to establish a monitoring system to detect and respond to social emotional flows related to food insecurity early by utilizing real-time text mining and sentiment analysis technologies based on unstructured data, such as online news, social media, and communities. In conclusion, food insecurity is a multidimensional emotional and sociocultural problem, and a single supply-oriented approach is not sufficient. As a result, it is judged that such text mining technology can become a key tool in designing food and health policies.

## 6. Limitation and Future Studies

This study has the following limitations and, based on these, we would like to suggest future research directions. First, the analysis was performed on unstructured text data generated from the South Korean websites Naver and Daum, and the possibility of sample bias cannot be ruled out, since data collection was conducted focusing on a specific keyword, namely “food insecurity”. In other words, documents that are relevant but not directly exposed to keywords may have been excluded from the analysis. Therefore, future studies need to ensure diversity and comprehensiveness through data collection that includes food shortages and nutritional imbalances in addition to food insecurity. In addition, various sources, such as Instagram, Facebook, and X, should be integrated to enable a more multidimensional analysis. Second, since an emotional network analysis focusing on consumer responses in South Korea was included, there may be some differences from consumer sentiment or food insecurity awareness in other cultures or countries. Accordingly, the generalizability of the research results is limited. Therefore, it will be necessary to compare and analyze various countries other than South Korea to identify differences in awareness of food insecurity, thereby deriving more universal and policy-related implications. Third, there is a temporal limitation. Since this study analyzed only one year of data from 2024, long-term trend changes or accumulated changes over several years since the pandemic may not have been sufficiently reflected. Therefore, time series analysis using at least three years of data will allow for a more precise understanding of long-term trends in consumer sentiment changes related to food insecurity. Fourth, text mining and sentiment analysis are useful for identifying correlations, but they have limitations in clearly identifying causal relationships between variables. Therefore, future research will need to connect the results of sentiment analysis with data on actual consumer behavior to empirically identify the relationship between emotions and behaviors. Finally, the study will need to conduct analyses based on demographically centered datasets to ultimately derive final results.

## Figures and Tables

**Table 1:** Survey of collected data.

Data	Channel	Section	2024
Food insecurity	Naver	Blog	26,160
		Cafe	7637
	Daum	Blog	4.455
		Cafe	3

**Table 2:** Narrative coding index.

Categories	2024
Food/nutrition	116
Cause/need	99
Sentimental/response	94
Connection/subject	70
Total	379

**Table 3:** Text mining of food insecurity.

Rank	Word	Frequency	TF-IDF	Rank	Word	Frequency	TF-IDF
1	Food	76,460	12,185.7	26	Many	10,491	20,975.36
2	Insecurity	72,776	4165.255	27	Problem	10,337	21,392.58
3	Health	65,009	46,365.19	28	Treatment	10,219	23,637.09
4	Intake	30,770	35,141.11	29	Improvement	9721	20,697.33
5	Stress	28,274	35,392.8	30	Nerve	9546	21,458.17
6	Help	26,470	32,280.54	31	Sleep	9490	22,932.89
7	Depression	22,867	27,802.1	32	Processed food	9449	19,569.05
8	Symptom	21,042	32,467.51	33	Brain	9287	21,736.66
9	Good	20,672	29,869.37	34	Exercise	8972	21,365.58
10	Mental	18,732	30,658.68	35	Stability	8796	19,649.24
11	Dish	16,644	29,469.45	36	Reduce	8698	18,866.3
12	Body	16,120	27,221.66	37	Balance	8583	19,564.36
13	Eat	15,694	29,457.07	38	People	8498	19,130.61
14	Function	15,404	26,893.31	39	Vegetable	8244	18,024.83
15	Effect	14,867	29,867.79	40	Inclusion	8005	17,812.78
16	Important	13,790	24,125.14	41	Mind	7933	18,692.32
17	Influence	13,035	24,156.91	42	Fruit	7734	17,442.91
18	Management	12,534	24,690.3	43	Decrease	7417	17,542.98
19	Method	12,224	23,477.33	44	Need	7266	16,897.16
20	Obstacle	11,495	24,423.79	45	Vitamin A	7149	22,085.36
21	Maintain	11,414	22,444.73	46	Mood	7125	17,479.93
22	Effectiveness	11,383	22,940.46	47	Cause	7059	18,074.13
23	Diet	11,311	22,888.57	48	Omega	6842	19,522.23
24	Relax	11,287	22,325.13	49	Causing	6706	16,489.62
25	Magnesium	10,507	29,402.93	50	Prevention	6701	16,809.99

**Table 4:** Sentimental network index of food insecurity.

Rank	Word	Degree Centrality	Betweenness Centrality	Closeness Centrality	Page Rank	Group	Category
1	Insecurity	209	26.727	0.005	1.077	1	Sentimental/response
2	Fatty acid	200	17.547	0.005	1.035	1	Food/nutrition
3	Vitamin D	199	14.912	0.005	1.030	1	Food/nutrition
4	Calcium	205	20.221	0.005	1.058	1	Food/nutrition
5	Fiber	198	13.411	0.005	1.025	1	Food/nutrition
6	Iron	196	15.450	0.005	1.017	1	Food/nutrition
7	Theanin	176	11.173	0.004	0.928	1	Food/nutrition
8	Melatonin	192	14.440	0.004	0.999	1	Food/nutrition
9	Oriental medicine	194	15.274	0.004	1.008	1	Food/nutrition
10	Salmon	198	14.924	0.005	1.026	1	Food/nutrition
11	Tryptophan	193	13.947	0.004	1.003	1	Food/nutrition
12	Vitamin C	196	17.604	0.005	1.018	1	Food/nutrition
13	Dairy product	200	16.169	0.005	1.035	1	Food/nutrition
14	Walnut	197	14.864	0.005	1.021	1	Food/nutrition
15	Spinach	195	13.656	0.004	1.012	1	Food/nutrition
16	Amino acid	199	20.392	0.005	1.032	1	Food/nutrition
17	Neurotransmitter	197	21.038	0.005	1.023	1	Food/nutrition
18	Antioxidant	201	15.937	0.005	1.039	1	Food/nutrition
19	Green tea	197	14.022	0.005	1.021	1	Food/nutrition
20	Yogurt	196	13.918	0.005	1.016	1	Food/nutrition
21	Help	209	26.727	0.005	1.077	1	Sentimental/response
22	Effect	209	26.727	0.005	1.077	1	Sentimental/response
23	Mood	209	26.727	0.005	1.077	1	Sentimental/response
24	Change	209	26.727	0.005	1.077	1	Sentimental/response
25	Psychology	209	26.727	0.005	1.077	1	Sentimental/response
26	Recommend	209	26.727	0.005	1.077	1	Sentimental/response
27	Side effect	209	26.727	0.005	1.077	1	Sentimental/response
28	Danger	209	26.727	0.005	1.077	1	Sentimental/response
29	Take effect	209	26.727	0.005	1.077	1	Sentimental/response
30	Positive	209	26.727	0.005	1.077	1	Sentimental/response
31	Insomnia	207	25.099	0.005	1.068	1	Sentimental/response
32	Worry	209	26.727	0.005	1.077	1	Sentimental/response
33	Postscript	209	26.727	0.005	1.077	1	Sentimental/response
34	Tiredness	206	24.362	0.005	1.063	1	Sentimental/response
35	Advantage	207	24.716	0.005	1.068	1	Sentimental/response
36	Solution	209	26.727	0.005	1.077	1	Sentimental/response
37	Poor	205	23.119	0.005	1.059	1	Sentimental/response
38	Negation	206	20.610	0.005	1.062	1	Sentimental/response
39	Reference	208	25.018	0.005	1.072	1	Sentimental/response
40	Caution	209	26.727	0.005	1.077	1	Sentimental/response
41	Food	209	26.727	0.005	1.077	2	Food/nutrition
42	Magnesium	207	24.936	0.005	1.068	2	Food/nutrition
43	Processed food	208	22.250	0.005	1.071	2	Food/nutrition
44	Vitamin B	209	26.727	0.005	1.077	2	Food/nutrition
45	Caffeine	204	18.882	0.005	1.053	2	Food/nutrition
46	Tonic	207	24.751	0.005	1.068	2	Food/nutrition
47	Sugar	203	17.791	0.005	1.048	2	Food/nutrition
48	Protein	207	24.661	0.005	1.068	2	Food/nutrition
49	Vitamin	208	25.657	0.005	1.072	2	Food/nutrition
50	Fermentation	206	19.628	0.005	1.062	2	Food/nutrition
51	Microorganism	197	15.635	0.005	1.021	2	Food/nutrition
52	Probiotics	199	16.772	0.005	1.030	2	Food/nutrition
53	Fish	205	18.612	0.005	1.057	2	Food/nutrition
54	Serotonin	207	25.590	0.005	1.068	2	Food/nutrition
55	Water	209	26.727	0.005	1.077	2	Food/nutrition
56	Coffee	204	18.876	0.005	1.053	2	Food/nutrition
57	Mineral	206	24.057	0.005	1.063	2	Food/nutrition
58	Egg	207	20.579	0.005	1.066	2	Food/nutrition
59	Additive	207	21.220	0.005	1.067	2	Food/nutrition
60	Medical supplies	200	22.748	0.005	1.037	2	Food/nutrition
61	Degenerate	207	21.879	0.005	1.067	2	Sentimental/response
62	Wellbeing	200	19.383	0.005	1.036	2	Sentimental/response
63	Purchase	206	20.747	0.005	1.062	2	Sentimental/response
64	Inconvenience	201	16.336	0.005	1.039	2	Sentimental/response
65	Production	201	22.779	0.005	1.041	2	Sentimental/response
66	Happy	206	20.148	0.005	1.062	2	Sentimental/response
67	Freedom	204	20.464	0.005	1.053	2	Sentimental/response
68	Limit	202	17.696	0.005	1.044	2	Sentimental/response
69	Interruption	202	17.565	0.005	1.044	2	Sentimental/response
70	Promote	205	23.607	0.005	1.059	2	Sentimental/response
71	Practice	194	13.540	0.004	1.008	2	Sentimental/response
72	Experience	156	8.347	0.004	0.840	2	Sentimental/response
73	Investment	168	9.922	0.004	0.893	2	Sentimental/response
74	Sharing	198	18.504	0.005	1.027	2	Sentimental/response
75	Efficiency	202	20.074	0.005	1.044	2	Sentimental/response
76	Expectation	162	7.288	0.004	0.866	2	Sentimental/response
77	Hard	202	23.206	0.005	1.045	2	Sentimental/response
78	Popularity	201	23.293	0.005	1.041	2	Sentimental/response
79	Action	184	14.275	0.004	0.964	2	Sentimental/response
80	Emphasis	191	16.610	0.004	0.995	2	Sentimental/response
81	Omega	204	17.602	0.005	1.053	3	Food/nutrition
82	Bean	205	18.900	0.005	1.057	3	Food/nutrition
83	Lactobacillus	197	15.713	0.005	1.021	3	Food/nutrition
84	Inositol	152	4.112	0.004	0.820	3	Food/nutrition
85	Enzyme	193	14.602	0.004	1.003	3	Food/nutrition
86	Salt	191	12.974	0.004	0.994	3	Food/nutrition
87	Organic farming	195	15.610	0.004	1.013	3	Food/nutrition
88	Potassium	185	11.319	0.004	0.967	3	Food/nutrition
89	Conquest	205	19.866	0.005	1.058	3	Sentimental/response
90	Liveliness	207	25.082	0.005	1.068	3	Sentimental/response
91	Removal	206	24.870	0.005	1.063	3	Sentimental/response
92	Perfection	202	19.208	0.005	1.044	3	Sentimental/response
93	Comfortable	193	15.814	0.004	1.004	3	Sentimental/response
94	Communication	176	9.570	0.004	0.927	3	Sentimental/response

**Table 5:** Time series analysis of key word frequencies.

Date	Food	Insecurity	Health	Intake	Stress	Mental	Body	Processed Food	Vegetable	Fruit
2024-01-1w	1388	1255	899	493	451	202	226	104	119	119
2024-01-3w	1475	1309	863	597	455	246	239	85	114	123
2024-01-5w	1366	1283	804	562	451	234	252	101	107	105
2024-02-2w	1368	1257	900	586	537	230	213	89	119	129
2024-02-4w	1328	1290	1048	510	534	288	302	209	140	132
2024-03-2w	1341	1299	990	489	541	273	266	175	128	116
2024-03-4w	1436	1307	1058	546	511	226	279	177	121	116
2024-04-2w	1346	1414	1029	610	485	272	284	185	141	137
2024-04-4w	1432	1337	964	496	535	232	233	153	110	116
2024-05-1w	1354	1300	930	583	555	267	295	147	124	118
2024-05-3w	1510	1337	866	534	483	292	259	126	118	111
2024-06-1w	1480	1362	1028	591	568	284	277	128	125	124
2024-06-3w	1492	1363	970	571	560	243	236	126	144	142
2024-07-1w	1345	1391	1219	538	570	369	288	207	159	157
2024-07-3w	1355	1414	1576	559	533	495	372	276	214	180
2024-07-5w	1257	1344	1900	501	468	575	448	320	212	200
2024-08-2w	1295	1430	1901	544	511	527	459	301	236	207
2024-08-4w	1469	1376	1472	616	563	485	375	185	184	167
2024-09-2w	1476	1374	1425	566	483	483	391	175	163	163
2024-09-4w	1482	1413	1296	583	558	415	350	175	169	148
2024-10-1w	1487	1368	1408	624	471	481	307	163	164	146
2024-10-3w	1563	1477	1666	662	497	545	386	187	174	170
2024-11-1w	1584	1367	1473	597	551	423	309	157	169	150
2024-11-3w	1579	1475	1118	622	523	314	237	196	158	141
2024-12-1w	1570	1522	1423	699	657	404	331	238	194	166
2024-12-3w	1709	1706	1445	791	763	404	325	236	192	188
2024-12-5w	687	778	600	468	314	114	97	130	91	82

**Table 6:** Sentiment analysis of food insecurity.

	Frequency	Frequency Percentage
Good feeling	98,959	37.52512
Joy	4992	1.89296
Interest	7964	3.019938
Positive total	111,915	42.43802
Sadness	27,726	10.54021
Disgust	3978	1.51
Fear	87,479	33.17
Pain	27,892	10.58
Anger	1938	0.73
Fright	2716	1.03
Negative total	151,799	57.56198
Total	163,714	100.0

## Data Availability

The original contributions presented in the study are included in the article, further inquiries can be directed to the corresponding author.
